# Influence of associated femoral head fractures on surgical outcomes following osteosynthesis in posterior wall acetabular fractures

**DOI:** 10.1186/s12891-022-05777-w

**Published:** 2022-09-01

**Authors:** Po-Ju Lai, Chih-Yang Lai, I-Chuan Tseng, Chun-Yi Su, Yi-Hsun Yu

**Affiliations:** 1 The Department of Orthopedic Surgery, Linkou Branch, Musculoskeletal Research Centre, Chang Gung Memorial Hospital, Chang Gung University, Taoyuan, Taiwan; 2 The Department of Orthopedic Surgery, Taoyuan Branch, Chang Gung Memorial Hospital, Chang Gung University, Taoyuan, Taiwan; 3 The Department of Orthopedic Surgery, Keelung Branch, Chang Gung Memorial Hospital, Chang Gung University, Taoyuan, Taiwan

**Keywords:** Acetabular fracture, Associated femoral head fracture, Osteosynthesis

## Abstract

**Background:**

To date, no study has compared the surgical outcomes between posterior wall acetabular fractures with and without associated femoral head fractures. Therefore, we evaluated whether an associated femoral head fracture increases the incidence of fracture sequelae, including post-traumatic osteoarthritis (PTOA) and osteonecrosis of the femoral head (ONFH), following osteosynthesis for posterior wall acetabular fractures.

**Methods:**

This retrospective clinical study enrolled 183 patients who underwent osteosynthesis for posterior wall acetabular fractures between 2009 and 2019 at a level-1 trauma center. The incidence of PTOA, ONFH, and conversion to total hip arthroplasty (THA) was reviewed.

**Results:**

The incidence of PTOA, ONFH, and conversion to THA following osteosynthesis were 20.2%, 15.9%, and 17.5%, respectively. The average time for conversion to THA was 18.76 ± 20.15 months (range, 1–82). The results for the comparison of patients with associated femoral head fractures and isolated posterior wall acetabular fractures were insignificant (PTOA: 27.3% vs. 15.7%, *p* = 0.13; ONFH: 18.2% vs. 14.3%, *p* = 0.58; conversion to THA: 20.4% vs. 15.7%, *p* = 0.52). Upon evaluating other variables, only marginal impaction negatively affected ONFH incidence (odds ratio: 2.90).

**Conclusions:**

Our methods failed to demonstrate a significant difference in the rate of PTOA, ONFH, or conversion to THA in posterior wall acetabular fractures with and without an associated femoral head fracture. Beyond femoral head fractures, the marginal impaction of the acetabulum could have led to early sequelae.

**Level of evidence:**

Level III

## Background

Fractures of the posterior wall are the most common types of acetabular fractures, accounting for approximately one-third of all acetabular fractures [1–4]. Although anatomical reduction and stable internal fixation are the goals of osteosynthesis, various issues, including concomitant hip dislocation, advanced age of a patient, marginal impaction of the posterior wall, and simultaneous fracture of the femoral head, may lead to early sequelae, including post-traumatic osteoarthritis (PTOA) and osteonecrosis of the femoral head (ONFH). These sequelae may affect the soft tissues around the hip joint, alter fascia mechanics [5], and cause pain and disability. Therefore, different studies report varied surgical outcomes for patients with posterior wall fractures; some studies report an unacceptably high rate of early conversion to total hip arthroplasty (THA) due to PTOA or ONFH [6–9].

Of these factors, whether the concomitant presence of a femoral head fracture (i.e., Pipkin type IV fracture) would increase the incidence of early ONFH remains debatable [10, 11]. Unfortunately, there is little evidence regarding this issue. Several studies have reported that isolated femoral head fractures can lead to ONFH [12–15]. However, most reports included all types of femoral head fractures, from Pipkin type I to IV.

To date, no study has compared the surgical outcomes between posterior wall acetabular fractures with and without associated femoral head fractures. Therefore, our study aimed to evaluate whether the presence of a femoral head fracture in posterior wall acetabular fractures would increase the rate of early PTOA or ONFH. Additionally, other potential factors that may increase the incidence of early sequelae were evaluated.

## Methods

We retrospectively reviewed all patients who underwent osteosynthesis for an acetabular fracture from a level 1 trauma center registry between 2009 and 2019. Patients with an elementary acetabular fracture pattern accompanying a posterior wall fracture were included, while those with a fracture pattern other than the posterior wall or a combined pelvic ring fracture were excluded. Additionally, patients aged < 18 years or who could not complete a one-year follow-up were excluded. This study was approved by the review board of our institution (IRB NO: 202101823B0).

### Resuscitation and perioperative treatment protocol

All patients followed the treatment protocol for acetabular fractures in our hospital. If a concomitant hip dislocation was present, immediate joint reduction was performed. Post-reduction, preoperative radiographic evaluation included an anteroposterior view and two Judet 45° oblique radiographs of the pelvis. Three-dimensional reconstructed computed tomography (CT) of the acetabulum was performed to evaluate the presence of intraarticular osteochondral fragments, marginal impaction, a fragment size of the posterior wall, and associated femoral head fracture for subsequent surgical planning. Definite osteosynthesis was performed when the patient’s general condition permitted the procedure.

Surgical approaches and implant selections were largely dependent on the fracture location. The Kocher–Langenbeck approach was used in simple posterior wall acetabular fractures. The fractures were reduced and fixed with pre-contoured reconstruction plates (DepuySynthes, Raynham, MA, USA). When marginal impaction of a fragment was present, the impacted osteochondral fragment was first disimpacted and reduced. Next, the void was filled with bone grafts and fixated with interfragment screws. Finally, the major posterior wall fragments were fixated.

For a patient with a Pipkin type IV fracture, the femoral head fragment was treated conservatively (small fragments and infrafoveal location with congruent femoral head contour) or surgically. The size, location, and existence of multi-fragments of the femoral head fracture were primarily evaluated by CT. Basically, we fixed all supra-fovea femoral head fractures because they involved the weight-bearing zone of the femoral head. For infra-fovea fractures, we only fixed them if they were large enough to cause incongruity or instability of the hip joint. For small pieces of fragments (the diameter of the applied screw is larger than the fragment), they were excised to avoid loose bodies within the hip joint. When the fracture was indicated for osteosynthesis, the posterior wall of the acetabulum and femoral head were managed using a modified Gibson approach, greater trochanteric osteotomy, and surgical femoral head dislocation to fix both fractures simultaneously.

### Rehabilitation and follow-up protocol

Postoperatively, a standard radiographic evaluation presenting three views of the pelvis was performed. The maximum displacement of the fracture seen at any view indicated the reduction quality of the posterior acetabular wall according to Matta’s reduction criteria: anatomical (displacement: 0–1 mm), imperfect (displacement: 2–3 mm), or poor (displacement: > 3 mm). Non-weight-bearing ambulation was advised for 4 weeks postoperatively, followed by 4 weeks of toe-touch weight-bearing ambulation, and then full weight-bearing was allowed. Functional and radiographic follow-ups were performed at 3 months, 6 months, 1 year, and annually thereafter.

### Definition of operation-related complications and sequelae

Surgery-related complications included perioperative vessel or nerve injury, early loss of reduction and fixation (< 3 months), and deep infection. Sequelae from the injury included PTOA and ONFH. PTOA was defined radiographically as a typical osteoarthritic change of the hip joint displaying joint space narrowing and osteophyte formation, with correlated clinical symptoms. ONFH was defined as a painful condition where the blood supply to the femoral head was disrupted, displaying radiographic changes of subchondral insufficiency or late femoral head collapse. The period between index surgery and THA was recorded if a patient underwent THA due to PTOA or ONFH.

### Subgroup analysis

Subgroup analysis was performed for patients with or without associated femoral head fractures. Patients with associated femoral head fracture were classified as Group F, and those with isolated posterior wall acetabular fracture were classified as Group A. Other variables, including concomitant hip dislocation, posterior wall marginal impaction, and posterior wall fragment size, were also used for the subgroup analysis. The incidence of early sequelae in patients with or without each risk factor was calculated.

### Statistical analysis

Data were analyzed using SPSS software (version 26.0; SPSS Inc., Chicago, IL, USA). Continuous variables were compared using Student’s t-test. Categorical variables were compared using the chi-squared and Fisher’s exact tests; *p* < 0.05 indicated statistical significance.

## Results

**Fig. 1:**
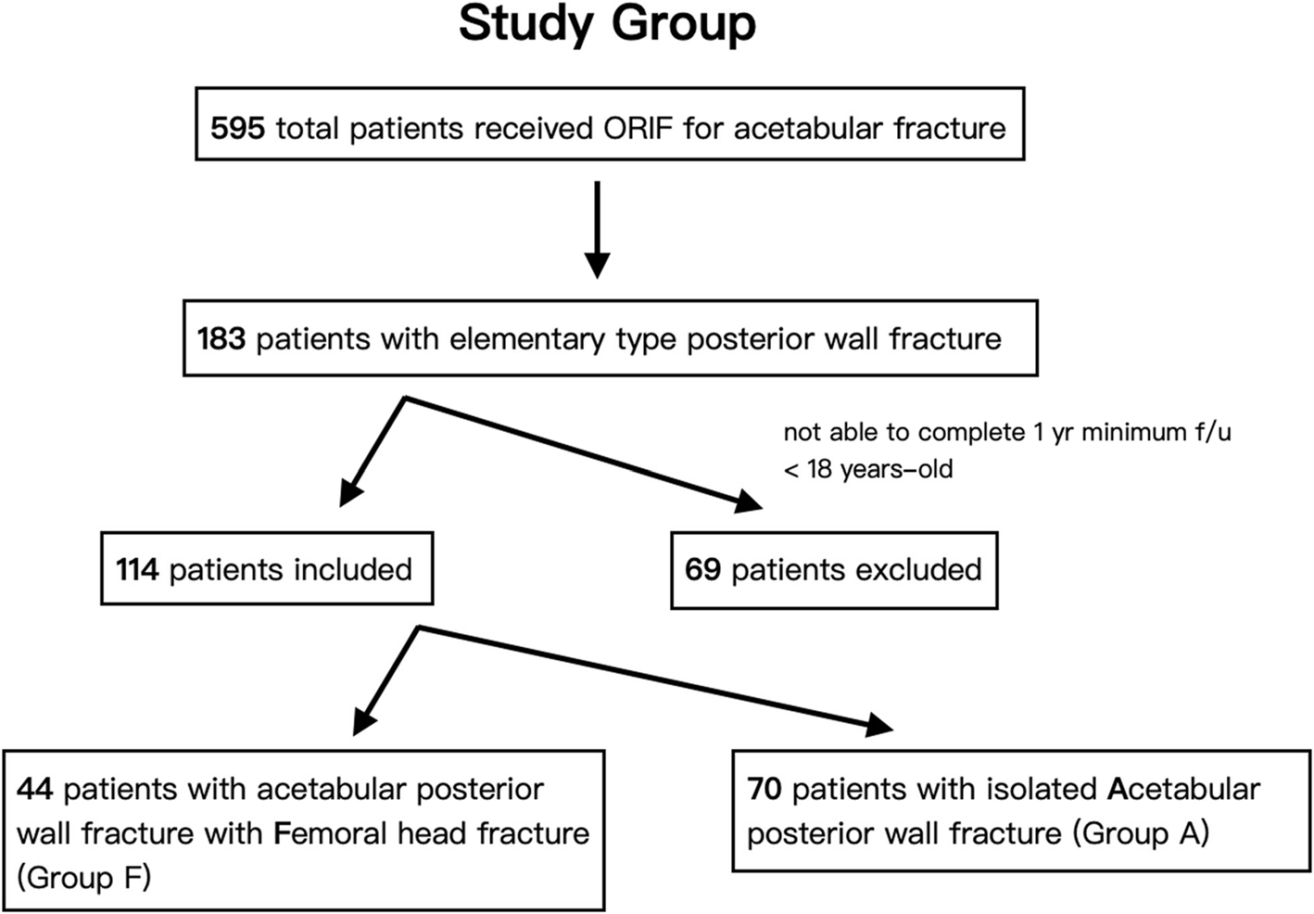
Study flowchart. The image illustrates the study group inclusion/exclusion tree

From 2009 to 2019, 595 patients underwent osteosynthesis for acetabular fractures in our hospital. Of these patients, 183 (30.8%) had posterior wall acetabular fractures, of which 114 patients (114/183, 62.3%) who met the minimum follow-up requirement (12 months) were included in this study (Fig. 1). The mean follow-up period for the study cohort was 41.07 months (range, 12–134). In this predominantly male (81.6%) cohort, the average age was 35.89 years, and the injury mechanism was primarily due to motorcycle collisions (77.2%) (Table 1). Hip dislocations were present in 106 patients (93.0%), and the average time for relocation of the hip joint was 4.92 h post-injury (range, 1–36). The average time from injury to osteosynthesis was 6.71 days (range, 0–30). Preoperative CT revealed marginal impaction in 42 of the total fractures (36.8%). The average size of the posterior wall fragment was 36.95%, calculated with Moed’s method [16]. The reduction quality of the posterior acetabular wall was graded as anatomical, imperfect, and poor in 102 (89.5%), 11 (9.6%), and 1 (0.09%) patients, respectively. Twenty-three patients (20.2%) were diagnosed with PTOA and eighteen (15.8%) with ONFH during follow-up. Twenty patients (17.5%) eventually underwent THA for the above conditions; the average time to THA was 18.76 months (range, 1–82).

**Table 1:** Demographic characteristics of patients with posterior wall acetabular fracture after surgical fixation and at least 1-year follow-up period between 2009 and 2019 at our institution

Number	114
Age (mean ± SD) years	35.89 ± 15.45
Sex (%)	
Male	93 (81.6%)
Female	21 (18.4%)
ISS (mean ± SD)	12.66 ± 7.71
NISS (mean ± SD)	19.45 ± 7.57
BMI (mean ± SD)	26.89 ± 5.21
Injury mechanism (%)	
MVA, motorcycle	88 (77.2%)
MVA, car	24 (21.0%)
Fall from height	2 (1.8%)
Marginal impaction (%)	42 (36.8%)
Posterior wall fragment size (Moed’s method) (mean ± SD) (%)	36.95 ± 20.93)
Hip dislocation (%)	106 (93.0%)
Reduction within 6 h	77 (72.6%)
Average time to reduction (mean ± SD) (hours)	4.92 ± 4.49
Time to osteosynthesis (mean ± SD) (days)	6.71 ± 5.19
Length of stay (mean ± SD) (days)	12.04 ± 8.08
Follow-up (mean ± SD) (months)	41.07 ± 29.37
Reduction quality	
Anatomical	102 (89.5%)
Imperfect or poor	12 (10.5%)
Perioperative complications (%)	
Vessel or nerve injury	3 (2.6%)
Early loss of reduction and fixation (< 3 months)	1 (0.9%)
Deep infection	5 (4.4%)
Fracture sequelae (%)	
PTOA	23 (20.2%)
ONFH	18 (15.8%)
Conversion to THA (%)	20 (17.5%)
Mean time to THA (mean ± SD) (months)	18.76 ± 20.15

*ISS* injury severity score, *NISS* new injury severity score, *BMI* body mass index, *MVA* motor vehicle accident, *PTOA* post-traumatic osteoarthritis, *ONFH* osteonecrosis of femoral head, *THA* total hip arthroplasty

We classified the whole cohort into Groups F and A according to the presence of a concomitant femoral head fracture. There were 44 patients in Group F and 70 patients in Group A. The mean age in Group F was significantly lower than that in Group A (32 vs. 38.33, *p* = 0.03). In addition, a significant difference was observed in the fragment size of the posterior wall, with that of Group F being significantly smaller (24.50 vs. 44.90, *p* = 0.00) than that of Group A. However, other demographic data showed no statistical difference (Table 2).

**Table 2:** Group analysis for acetabular posterior wall fracture with femoral head fracture (Group F) and isolated acetabular posterior wall fracture (Group A)

	Group F	Group A	*p*-value
Number	44	70	
Age (mean ± SD) years	32 ± 15.45	38.33 ± 15.05	0.03
Sex (%)			0.96
Male	36 (81.4%)	57 (81.8%)	
Female	8 (18.6%)	13 (18.2%)	
ISS (mean ± SD)	11.55 ± 6.30	13.36 ± 8.44	0.19
NISS (mean ± SD)	19.88 ± 5.86	19.17 ± 8.50	0.60
BMI (mean ± SD)	26.93 ± 4.55	26.87 ± 5.65	0.96
Injury mechanism (%)			0.88
MVA, motorcycle	33 (75.0%)	55 (78.6%)	
MVA, car	10 (22.7%)	14 (20.0%)	
Fall from height	1 (2.3%)	1 (1.4%)	
Marginal impaction (%)	14 (31.8%)	28 (40%)	0.38
Posterior wall fragment size (Moed’s method) (mean ± SD) (%)	24.50 ± 18.88	44.90 ± 18.15	0.00
Hip dislocation (%)	43 (97.7%)	63 (90%)	0.15
Reduction within 6 h	30 (69.8%)	47 (74.6%)	0.74
Time to reduction (mean ± SD) (hours)	4.52 ± 4.18	5.19 ± 4.74	0.51
Time to osteosynthesis (mean ± SD) (days)	7.14 ± 6.26	6.44 ± 4.42	0.52
Length of stay (mean ± SD) (days)	12.02 ± 8.88	12.04 ± 7.61	0.99
Follow-up (mean ± SD) (months)	46.57 ± 35.56	37.61 ± 25.24	0.14

*ISS* injury severity score, *NISS* new injury severity score, *BMI* body mass index, *MVA* motor vehicle accident, *SD*, standard deviation

Perioperative comparisons between the two groups are shown in Table 3. Although there was a trend of more surgical time and greater blood loss during osteosynthesis in managing both acetabular and femoral head fractures, both were insignificant (*p* = 0.06 and 0.23, respectively). The reduction quality also showed no significant difference between the two groups, as the anatomical reduction was achieved in most cases (40/44, 90.9% vs. 62/70, 88.6%, *p* = 0.69).

**Table 3:** Group comparison for surgical method, reduction quality, and perioperative complications

	Group F	Group A	*p*-value
Femoral head fracture			
Fixation with screws	23 (52.3%)	-	
Fragment excision/no treatment	21 (47.7%)	-	
Surgical time (mean ± SD) (minutes)	171.98 ± 61.27	151.10 ± 49.44	0.06
Estimated blood loss (mean ± SD) (mL)	386.36 ± 258.39	307.86 ± 196.47	0.23
Reduction quality			0.69
Anatomical	40 (90.9%)	62 (88.6%)	
Imperfect or poor	4 (9.1%)	8 (11.4%)	
Perioperative complications (%)			
Vessel or nerve injury	2 (4.5%)	1 (2.3%)	0.55
Early loss of reduction and fixation	1 (2.3%)	0 (0%)	0.38
(< 3 months) Deep infection	2 (4.5%)	3 (4.3%)	0.95

Reduction quality was based on Matta’s reduction criteria

*SD* standard deviation

Moreover, the comparison of fracture sequelae after osteosynthesis between Groups F and A was insignificant (PTOA: 12/44, 27.3% vs. 11/70, 15.7%, *p* = 0.13 and ONFH: 8/44, 18.2% vs. 10/70, 14.3%, *p* = 0.58) (Table 4). In Group F, 20.4% of the patients eventually underwent THA during the follow-up period. However, these data were insignificant compared to that of Group A, wherein 15.7% of patients underwent THA (*p* = 0.52). Meanwhile, we further separated patients treated with or without femoral head osteosynthesis, and the fracture pattern was simple or multi-fragmentary in Group F (Table 5). There was no significant difference in the incidence of fracture sequelae in this group.

**Table 4:** Group comparisons for fracture sequelae after osteosynthesis

	Group F	Group A	*p*-value
Fracture sequelae			
PTOA	12 (27.3%)	11 (15.7%)	0.13
Mean time to PTOA (mean ± SD)	17.5 ± 7.77	19.0 ± 13.75	0.79
(months)	8 (18.2%)	10 (14.3%)	0.58
ONFH Mean time to ONFH (mean ± SD) (months)	8.67 ± 10.88	8.60 ± 7.69	0.99
Conversion to THA (%)	9 (20.4%)	11 (15.7%)	0.52
Mean time to THA (mean ± SD) (months)	15.78 ± 17.34	21.91 ± 23.67	0.51

*PTOA* post-traumatic osteoarthritis, *ONFH* osteonecrosis of femoral head, *THA* total hip arthroplasty, *SD* standard deviation

**Table 5:** Fracture sequelae in patients who received different treatments for femoral head fracture in Group F

	Fixation with screws		Fragment excision/no treatment	*p*-value
Patients with femoral head fracture (*n* = 44)	23		21	
PTOA	4 (17.4%)		8 (38.1%)	0.23
ONFH	5 (21.7%)		3 (14.3%)	0.80
Conversion to THA	4 (17.4%)		5 (23.8%)	0.88
	Simple fracture	Multi-fragmentary		
Patients received ORIF for femoral head fracture (*n* = 23)	17	6		
PTOA	3 (17.6%)	1 (16.7%)		1.00
ONFH	3 (17.6%)	2 (33.3%)		0.58
Conversion to THA	2 (11.8%)	2 (33.3%)		0.27

*PTOA* post-traumatic osteoarthritis, *ONFH* osteonecrosis of femoral head, *THA* total hip arthroplasty, *SD* standard deviation

In addition to simultaneous femoral head fracture, we evaluated previously reported factors possibly related to fracture sequelae in posterior wall acetabular fractures. These included concomitant hip dislocation and posterior wall marginal impaction. In our cohort, patients with or without concomitant hip dislocation showed no difference in the incidence of fracture sequelae, including PTOA (18.9% vs. 37.5%, *p* = 0.42), ONFH (16.0% vs. 12.5%, *p* = 0.79), and conversion to THA (17.9% vs. 12.5%, *p* = 0.69). In patients with concomitant hip dislocation, there was no difference between patients who received reduction within 6 h and those who did not (Table 6). Patients with marginal impaction had a higher rate of ONFH than patients without marginal impaction (23.8% vs. 9.7%, odds ratio [OR]: 2.90, *p* = 0.04) (Table 7). This was also true in Group A (28.6% vs. 4.8%, OR: 8, *p* = 0.01), but not in Group F (14.3% vs. 16.7%, *p* = 0.84). The impact of marginal impaction on the incidence of PTOA or conversion to THA was insignificant in the whole cohort and each subgroup. However, with the co-existence of femoral head fracture and marginal impaction of the femoral head, 35.7% of the patients had sequelae of PTOA (Table 8).

**Table 6:** Fracture sequelae in patients with or without concomitant hip dislocation

	Patients with hip dislocation	Patients without hip dislocation	*p*-value
All cohort (*n* = 114)	10,620 (18.9%)	83 (37.5%)	0.42
PTOA	17 (16.0%)	1 (12.5%)	0.79
ONFH Conversion to THA	19 (17.9%)	1 (12.5%)	0.69
	Reduction within 6 h	Reduction beyond 6 h	
Patients with hip dislocation (*n* = 106)	77	29	
PTOA	14 (18.2%)	6 (20.7%)	0.98
ONFH	12 (15.6%)	5 (17.2%)	0.84
Conversion to THA	14 (18.2%)	5 (17.2%)	0.91

*PTOA* post-traumatic osteoarthritis, *ONFH* osteonecrosis of femoral head, *THA* total hip arthroplasty,

**Table 7:** Fracture sequelae in patients with or without posterior wall marginal impaction

	With marginal impaction	Without marginal impaction	*p*-value
Group A (*n* = 70)	28	42	
PTOA	4 (14.3%)	7 (16.7%)	0.79
ONFH	8 (28.6%)	2 (4.8%)	0.01
Conversion to THA	7 (25.0%)	4 (9.5%)	0.11
Group F (*n* = 44)	14	30	
PTOA	5 (35.7%)	7 (23.3%)	0.39
ONFH	2 (14.3%)	5 (16.7%)	0.84
Conversion to THA	3 (21.4%)	6 (20.0%)	0.91
All cohort (*n* = 114)	42	72	
PTOA	9 (21.4%)	14 (17.1%)	0.80
ONFH	10 (23.8%)	7 (9.7%)	0.04
Conversion to THA	10 (23.8%)	10 (12.2%)	0.18

*PTOA* post-traumatic osteoarthritis, *ONFH* osteonecrosis of femoral head, *THA* total hip arthroplasty

**Table 8:** Incidence of fracture sequelae in patients with risk factors

Femoral head fracture	Marginal impaction	PTOA	ONFH
Yes	Yes	35.7%	14.3%
Yes	No	23.3%	16.7%
No	Yes	14.3%	28.6%
No	No	16.7%	4.8%

*PTOA* post-traumatic osteoarthritis, *ONFH* osteonecrosis of femoral head

We also analyzed the size difference in the posterior wall fragment between patients who suffered from fracture sequelae and those who did not. Further, no significant difference was observed between patients with and without PTOA, ONFH, or conversion to THA (39.16 vs. 36.39, *p* = 0.58; 39.21 vs. 36.52, *p* = 0.66; 39.34 vs. 36.47, *p* = 0.62) (Table 9).

**Table 9:** Relationship of posterior wall fragment size with fracture sequelae

Posterior wall fragment size (Moed’s method) (%)	In patients with …	In patients without	*p*-value
All cohort (*n* = 114)			
PTOA	39.16 (SD 21.53)	36.39 (SD 20.87)	0.58
ONFH	39.21 (SD 23.98)	36.53 (SD 20.43)	0.66
Conversion to THA	39.34 (SD 23.27)	36.47 (SD 20.53)	0.62

*PTOA* post-traumatic osteoarthritis, *ONFH* osteonecrosis of femoral head, *THA* total hip arthroplasty

## Discussion

Despite the relatively simple radiographic appearance of posterior wall acetabular fractures, they have a high rate of poor outcomes, even when treated by the most experienced surgeons. This is because the long-term outcome of osteosynthesis is interfered by the development of fracture sequelae, including PTOA and ONFH. Moreover, the reported posterior wall fracture sequelae incidence was 4–29% for PTOA and 5–12% for ONFH [7, 17–19]. Consequently, 20% of patients eventually undergo THA during long-term follow-up [9, 20–23]. In our cohort, the incidence was 20.2% for PTOA and 15.8% for ONFH, and 17.5% of patients required THA, similar to the results of previous reports. For patients who eventually develop end-stage osteoarthritis and receive THA after acetabular fractures, the stability of prosthesis and bone loss continue to pose challenges. Although there are several modern prostheses that provide solutions to such challenges [24], hip preservation continues to be the goal for the treatment of acetabular fracture.

According to published studies, several risk factors could be related to the sequelae of acetabular fractures. Our study focused on the influence of simultaneous femoral head fractures. Poletti et al. showed that femoral head subchondral impaction on CT was related to conversion to THA [25]. Rollmann et al. reported that femoral head contusion is related to PTOA and increases the likelihood of THA by 3.68 times in their pelvic registry study [22]. Tannast et al. designed a nomogram to predict the need for THA within 2 years. Femoral head cartilage lesions are the third most important factor behind patient age and reduction quality [26]. However, these studies described femoral head “impaction” or “chondral injury” rather than femoral head “fracture.” In studies on femoral head fractures, including Pipkin type I to IV, the incidence of ONFH ranged from 8.7% to 25% [27–29]. These studies demonstrated that damage to the proximal femur blood supply could occur at the initial injury or during the surgical procedure, especially with the posterior approach [30]. Therefore, we expected the outcomes of Group F to be worse than those of Group A, owing to a femoral head fracture. However, our methods failed to demonstrate a significant difference in the rate of fracture sequelae between both groups.

We attribute the satisfactory outcomes of our patients with simultaneous femoral head fractures to the utilization of trochanteric osteotomy. Trochanteric osteotomy with surgical hip dislocation provided better visualization of the anterosuperior aspect of the femoral head without further jeopardizing the blood supply [31–33]. In addition, this method decreased the possibility of PTOA and ONFH by avoiding extensive dissection and achieving adequate reduction at the weight-bearing site, respectively. In our cohort, the risk of fracture sequelae following osteosynthesis was similar in posterior wall acetabular fractures, regardless of the presence of a simultaneous femoral head fracture.

Since we identified that a concomitant femoral head fracture might not increase the incidence of fracture sequelae, other risk factors were evaluated. In concomitant hip dislocations, disruption of the blood supply during the dislocation could lead to ONFH [34]. However, there was no increase in the rate of ONFH following osteosynthesis in patients with initial hip dislocations in our study. Prompt relocation of the dislocated joint might explain this outcome. The average time to hip relocation was 4.92 h, within the advised cut-off point of 12 h by Moed et al. [35] or 6 h by Hougaard et al. [36]. Additionally, most of our cases presented with initial hip dislocations (93.0%). This uneven distribution might mask the actual effect of the initial hip dislocation on posterior wall acetabular fractures.

Marginal impaction is another risk factor that causes surgical difficulty and increases the risk of non-anatomical reduction during osteosynthesis, which is associated with PTOA [37–40]. Moreover, it has long been recognized as a risk factor perpetuating poor outcomes in posterior wall acetabular fractures [40–42]. Additionally, even if the impacted fragment was initially reduced, inadequate grafting can cause a secondary collapse of the articular surface and joint incongruity. However, we did not observe an increase in PTOA in patients with marginal impaction in our cohort. This could be due to the requirement of anatomically disimpacting osteochondral fragments and the improvement of the bone grafting technique [43, 44].

Conversely, we observed that patients with marginal impaction of the acetabulum had a higher incidence of ONFH than those without an impacted injury, and most of these patients were in Group A. Although there is no clear explanation for this finding, we postulate that a higher pressure on the femoral head could exist during injury in those with direct impact on the acetabulum than in those where the femoral head “slides” posteriorly, causing a posterior wall fracture without impacted fractures. Subsequently, a higher pressure received during the injury could cause ONFH.

Our result showed that the fragment size of the posterior wall was approximately two-fold higher in Group A than in Group F. Therefore, it is reasonable to infer that the force was partly shared by the femoral head in Pipkin type IV fractures. The impaction resulted in a smaller posterior wall fracture before hip dislocation as the size of the femoral head reduced. However, the posterior wall fragment size does not interfere with the treatment outcome, according to Shah et al. [45]. Additionally, similar findings were observed in our study, as no significant difference was observed in the posterior wall fragment size between patients with and without fracture sequelae.

Our study had several limitations. First, the data collection was retrospective. During the review of the 10-year-period cases, 37.7% of patients were excluded because of shorter follow-up time. The reason for a relatively high drop-out rate was that 52.3% of patients were referred to our hospital for surgery. After completing the treatment course, they were routinely followed up at the nearby hospital. This might have affected the statistical power of our study. Second, the follow-up duration might not be long enough to define the actual PTOA incidence. However, PTOA incidence following posterior wall acetabular fractures ranged from 4 to 29%, with variable follow-up durations. Third, we emphasized the rapid development of osteoarthritis within 1 year following trauma, with an incidence of 7.9%. A longer follow-up period should be examined to determine the actual incidence of PTOA in this cohort. Fourth, chondral lesions of the femoral head were detected in most cases treated surgically by direct visualization intraoperatively. For those considered for conservative treatment (no femoral head fractures on CT scan), we could not differentiate whether there was cartilage damage or not. This might have caused the bias in underestimating the importance of chondral injuries. Finally, the image follow-ups were radiographically evaluated instead of using a post-osteosynthesis CT scan to evaluate the reduction quality. Postoperative CT scans were not routinely performed until 2017 in our hospital. Therefore, radiographic images were used for evaluation to minimize bias and avoid using different evaluation tools to quantify reduction. Further studies should use CT scans as an imaging tool to determine the quality of reduction for such sophisticated intraarticular injuries.

## Conclusions

Our methods failed to demonstrate a significant difference in the rate of PTOA, ONFH, or conversion to THA in posterior wall acetabular fractures with and without an associated femoral head fracture. Beyond a femoral head fracture, the marginal impaction of the acetabulum seemed to be a predictive factor that led to early sequelae.

## Data Availability

The datasets used and/or analyzed during the current study are available from the corresponding author on reasonable request.

## Abbreviations

PTOA: Post-traumatic osteoarthritis
ONFH: Osteonecrosis of the femoral head
THA: Total hip arthroplasty
CT: Computed tomography
